# Reduction of virus isolation effectiveness from throat swab double positive to H1N1pdm09 and H3N2 influenza A virus strain with SARS-CoV-2 virus in cell cultures

**DOI:** 10.1007/s11033-026-11764-4

**Published:** 2026-04-03

**Authors:** Rosaria Arvia, Valentina Tassone, Simone Giannecchini

**Affiliations:** 1 Department of Experimental and Clinical Medicine, University of Florence, Florence, Italy; 2 Dipartimento di Medicina Sperimentale e Clinica, Università di Firenze, Viale Morgagni 48, Firenze, I-50134 Italy

**Keywords:** Influenza virus, SARS-CoV-2, Virus isolation, Virus interference, Co-infection, Cytokine mRNA expression

## Abstract

**Background:**

Influenza virus isolation in cell cultures can be a foremost activity in the surveillance of the epidemiological characteristics involved in the vaccination strategies.

**Methods and results:**

Here, the virus isolation effectiveness from double positive throat swab to influenza A virus (H1N1pdm09 or H3N2) and SARS-CoV-2 compared to those obtained from single positive throat swab samples was investigated by serial passages of throat swabs samples in cell cultures. Viral replication and IFN-α, IL-1α, and IL-6 cytokine expression at three passages were measured by RT-PCR amplification. Virus isolation using a throat swab samples resulted double positive to influenza A virus (H1N1pdm09 or H3N2) and SARS-CoV-2 showed a different effectiveness compared to a throat samples single positive to each of these viruses. The virus isolation efficacy was independent from an active replication of the two viruses in the inoculum and showed a virus strain-specific reduction in cytokines profile compared to a single infection.

**Conclusion:**

Collectively the results point out to take into account the presence of co-circulating respiratory viruses in human samples for virus isolation effectiveness.

## Introduction

Respiratory viruses, including enveloped and naked viruses with DNA and RNA genomes, are common pathogens that cause from mild to severe illnesses worldwide each year [1]. Sometimes simultaneous infections by more than one respiratory virus can occur, which usually leads to increased disease severity, diagnostic difficulties, and stress on the immune response [2, 3]. To date, probability of a simultaneous infections with the high circulation of influenza viruses coupled with new emerged severe acute respiratory syndrome coronavirus 2 (SARS-CoV-2) is a main concern for public health [4]. The interaction of these respiratory viruses and the role of cytokines during simultaneous infection is controversial [5, 6]. Co-infection with influenza and SARS-CoV-2 is associated with significantly worse clinical outcomes, including higher mortality and increased need for intensive care [4]. Moreover, from a diagnostic point of view, coinfection of these respiratory viruses can hampers the efficiency of virus isolation techniques performed routinely to analyze virus threat for the epidemiology and vaccinology features. During the last influenza season (2024/25), among all positive throat swab samples examined within routine respiratory virus diagnostic and surveillance activities, we collected one throat swab double positive for influenza A(H1N1)pdm09 and SARS-COV-2 and other one double positive for influenza A(H3N2) and SARS-COV-2 (all detected by virus type specific RT-real time-PCR amplification technique). It was observed a difficulty to isolate from double positive throat swab samples and to cultivate effectively the two strains of influenza viruses in Madin-Darby canine kidney (MDCK) cell line and the strain of SARS-CoV-2 in African green monkey kidney epithelial cells (VERO E6) cell line, compared to those obtained from single positive throat swab samples. This observation encouraged us monitoring viral replication during their three consecutive passages in cell substrates. Moreover, it was considered of interest analyze mRNA expression of three cytokines, interferon-alpha (IFN-α), interleukin 1 alpha (IL-1 α) and interleukin-6 (IL-6), which are involved in the regulation of the immune response against respiratory viruses [7, 8].

## Materials and methods

### Cells and throat swab samples

MDCK (Madin-Darby canine kidney, CCL-34 Rockville, Md, USA) was cultivated using Minimum Essential Medium (MEM, Merck, Darmstadt, Germany) supplemented with 10% fetal bovine serum (FBS, Merck, Darmstadt, Germany). VERO E6 (CRL-1586, ATCC, Rockville, Md, USA) and A549 (adenocarcinomic human alveolar basal epithelial cells, ATCC CCL-185, Rockville, Md, USA) were cultivated using Dulbecco’s Modified Eagle Medium (DMEM, Merck, Darmstadt, Germany) supplemented with 10% FBS. All cellular substrates were obtained from stock of frozen cells routinely to maintain high susceptibility of respiratory virus isolation. Throat swabs, collected during epidemical season 2024-25, double positive for influenza virus A/H1N1pdm09 (real-time PCR diagnostic positive by cycle threshold (Ct) 20 values) and SARS-CoV-2 (sample tested real-time PCR diagnostic positive with a Ct 28 values) (sample 1) and double positive for influenza virus A/H3N2 (sample tested real-time diagnostic positive with a Ct 28 values) and SARS-CoV-2 (sample tested real-time PCR diagnostic positive with a threshold cycle Ct 27 values) (sample 2) were selected. Additionally, throat swab sample positive for single influenza virus A(H1N1)pdm09 infection (sample tested real-time PCR diagnostic positive with a threshold cycle Ct 23 values) (sample 3), for single influenza virus A(H3N2) infection (sample tested real-time PCR diagnostic positive with a threshold cycle Ct 23 values) (sample 4), and for single SARS-CoV-2 infection (sample tested real-time PCR diagnostic positive with a Ct 25 values) (sample 5) were used as control (Table 1).

**Table 1:** Throat swab samples used in the study

Sample	Virus positivity detected	Real-time PCR cycle threshold	ID in the figure
1	A/H1N1pdm09 and SARS-CoV-2	Ct 20 (A/H1N1pdm09); ct 28 (SARS-CoV-2)	H1-SARS-CoV-2; SARS-CoV-2-H1
2	A/H3N2 and SARS-CoV-2	Ct 28 (A/H3N2); ct 27 (SARS-CoV-2)	H3-SARS-CoV-2; SARS-CoV-2-H3
3	A/H1N1pdm09	Ct 23	H1
4	A/H3N2	Ct 23	H3
5	SARS-CoV-2	Ct 25	SARS-CoV-2

### Infection experiments

MDCK, VERO E6 and A549 cells were counted with trypan blue in Burker camera and seeded in 24-well plates at a density of 2.5 × 10^4^ cells per well and incubated overnight at 37°C in a 5% CO2 atmosphere to allow adhesion and reach approximately 80% confluence. The next day, the cells were infected with sample 1, sample 2, sample 3, sample 4 and sample 5 using the same viral genome yield estimated normalizing the real-time ct values obtained during diagnostic outcome for each virus. Each inoculum used was prepared by diluting the clinical sample in 100 µL of serum-free MEM or DMEM and added to the cells. In particular, each of three single positive samples were normalized to contain same specific viral genome ct values present in double infected samples. The cells were incubated for 1 h at 37°C in a 5% CO2 atmosphere; then, the inoculum was removed and the cells were washed twice with PBS to remove unbound virus. Subsequently new serum-free MEM or DMEM supplemented with L-1-tosylamido-2-phenylethyl-chloromethyl-ketone-treated trypsin (2 µg/mL; Sigma, St. Louis, MO, USA) was added to the cells and incubated at 37°C in a 5% CO2 atmosphere for 72 h. Then viral RNA was extracted from each inoculum and from the cells A549. Although not mandatory, all virus isolation experiments were performed at the time of this study under biosafety level-3 (BSL3) containment.

### RNA extraction and one-step RT real-time PCRs

Total RNA was extracted from 150 µL of throat swab fluid or cells supernatant using the QIAamp RNA mini Kit (Qiagen, Hilden, Germany) and from infected cells A549 using the RNAeasy mini kit (Qiagen, Hilden, Germany) according to the manufacturer’s instructions. AgPath-ID One-Step RT-PCR (Applied Biosystems, Waltham, MA, USA) was used for each RT-real time-PCR amplification according to the manufacturer’s instructions, using primers targeting the H1 gene of H1N1pdm09 Influenza A virus (primer forward H1pdm-For Primer GTG CTA TAA ACA CCA GYC TCC CAT T, primer reverse H1pdm-Rev Primer AGA YGG GAC ATT CCT CAA TCC TG and probe H1pdm-Probe Fam – 5’ TGG CCA GYC “T” CA ATT TTG TGC TTT TTA CAT A − 3’ – BHQ-1), the H3 gene of H3N2 Influenza A virus (primer forward H3-For Primer AAG CAT TCC MAA TGA CAA ACC, primer reverse H3-Rev Primer CAT YCC TGT TGC CAA TTT CAG and probe H3-Probe Fam – 5’ CAG GAT CAC/Nova/ATA YGG GGC MTG TCC CAG – 3’– BHQ-1) and using primers targeting the N gene of SARS-CoV-2 (primer forward SC2-For 5’ CTG CAG ATT TGG ATG ATT TCT CC 3’, reverse SC2-Rev 5’ CCT TGT GTG GTC TGC ATG AGT TTA G 3’ and probe SC2-Probe FAM-5’ ATT GCA ACA ATC CAT GAG CAG TGC TGA 3’-MGB. The reactions were performed in duplicate on Rotor-Gene Q real-time instrument (Qiagen, Hilden, Germany).

### Cytokine mRNA expression studies

At 72 h post-infection for three time passages (p1, p2 and p3), mRNA of A549 were analyzed to study the expression of IL-1α, IFN-α and IL-6 by comparative RT-PCRs using validated PrimePCR SYBR Green Assays (Bio-Rad, Hercules, CA, USA). Expression of the selected genes in three independent infected cultures was calculated using the ΔΔCt method and the results were displayed as fold change relative to mock-infected culture (infected culture vs. uninfected culture) and as fold change relative to infected single virus (infected with throat swab virus double positive vs. single virus positive). The expression of target genes was normalized to the expression of the 18 S gene. Data were analyzed using a two-tailed *Student’s t-test*. Differences were considered statistically significant when *p* < 0.05.

## Results and discussion

### Monitoring viral passages in MDCK, VERO E6 and A549

**Fig. 1:**
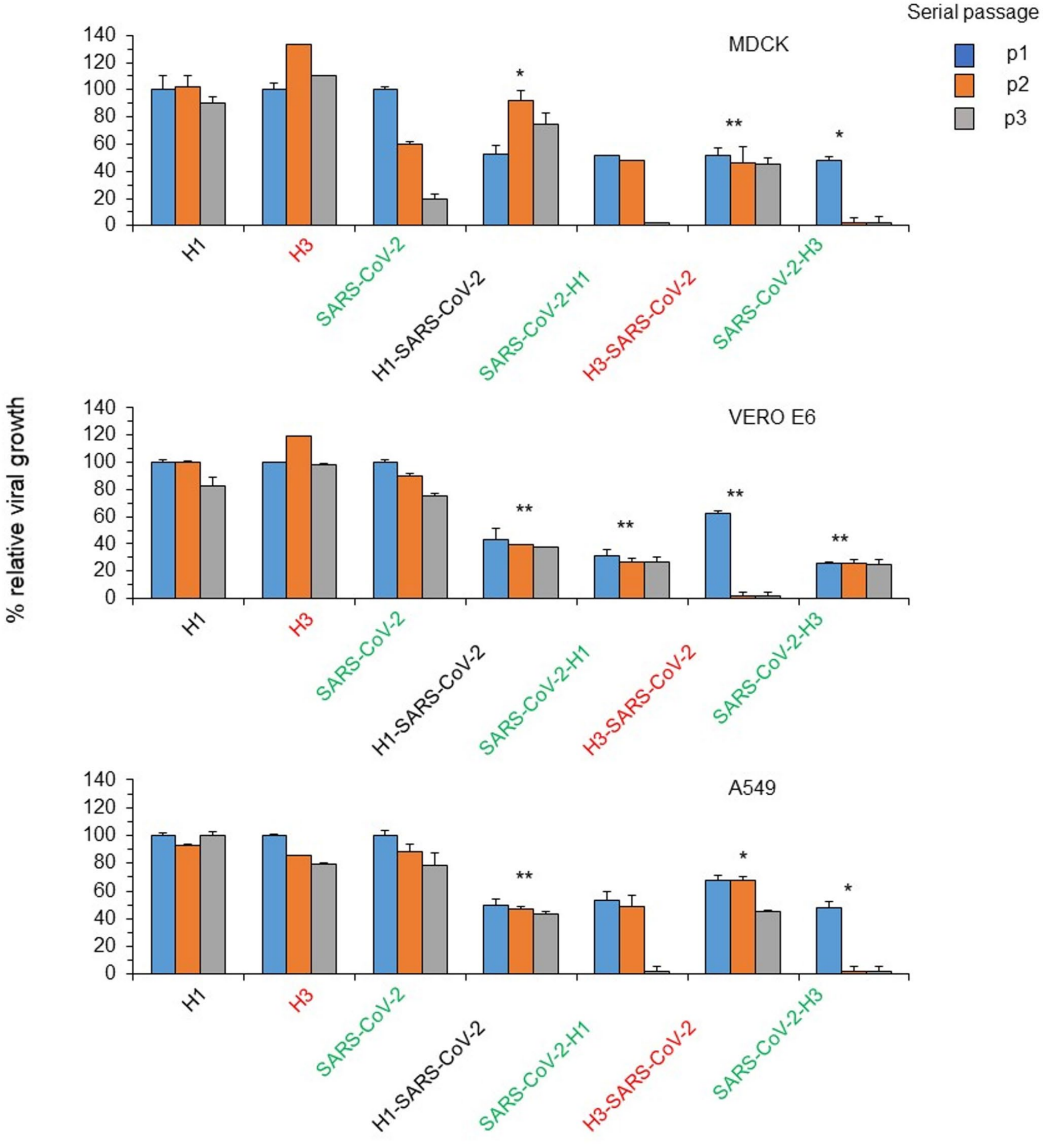
Virus infection serial passages history performed with double and single positive throat swab samples on different cellular substrate of replication. MDCK, VERO E6 and A549 cells were infected with respiratory samples double and single positive at indicated viruses using normalized Ct genomic values. After first passage, supernatant of infected cells was collected after 72 h post infection and used to perform second and third serial passages. At each passage, supernatant was used to extract RNA. One hundred nanograms of total RNA were amplified using primer and probes specific for H1N1pdm09, H3N2 and SARS-CoV-2 genomic region as reported in the text. The amplification of specific viral target in coinfection is indicated by different color (black, red and green) used for related virus in single infection. The kinetic of viral growth was obtained comparing ct values of each virus used in coinfection to ct values of same virus used in single infection at each passage. Values shown are mean + standard deviation obtained in 3 independent experiments. *p < 0.05, **p < 0.01, *student’s t-test.*

The isolation and subsequent passages of single and double positive throat swab samples (Table 1) were performed on MDCK, VERO E6, and A549 cell line. In MDCK, as shown in Fig. 1, examining the molecular gene target for each virus relative expression, the single H1N1pdm09 and H3N2 positive sample (samples 3 and 4) showed a significantly higher viral replication kinetics than those obtained using double H1N1pdm09/SARS-CoV-2 and H3N2/SARS-CoV-2 double positive samples. Furthermore, the cytopathic effect was more evident in mono-infection than in co-infection. In this experiment, the impaired virus replication observed using SARS-CoV-2 single positive sample confirm the MDCK restriction to this virus replication. Then, it was repeated the experiment using VERO E6 cells as a susceptible cellular substrate for SARS-CoV-2 replication. Figure 1 confirmed that the viral replication of H1N1pdm09 and H3N2 using double positive samples (samples 1 and 2) results significantly impaired compared those obtained with throat samples (samples 3 and 4) with a single virus especially for H3N2 virus that was completely lost at the third passages. The viral replication of SARS-CoV-2 using double positive samples (samples 1 and 2), also, results significantly impaired compared those obtained with single positive throat sample (sample 5).

Lastly, the viral replication was analyzed in the A549 cells line as a cell substrate highly susceptible for each virus. In this context, in infection performed with a double positivity for H1N1pdm09/SARS-CoV-2 and H3N2/SARS-CoV-2 the viral replication kinetics of H1N1pdm09 and H3N2 was reduced during the three sequential passages, while the viral replication kinetics of SARS-CoV-2 was abolished.

### Cytokine mRNA expression profiles

**Fig. 2:**
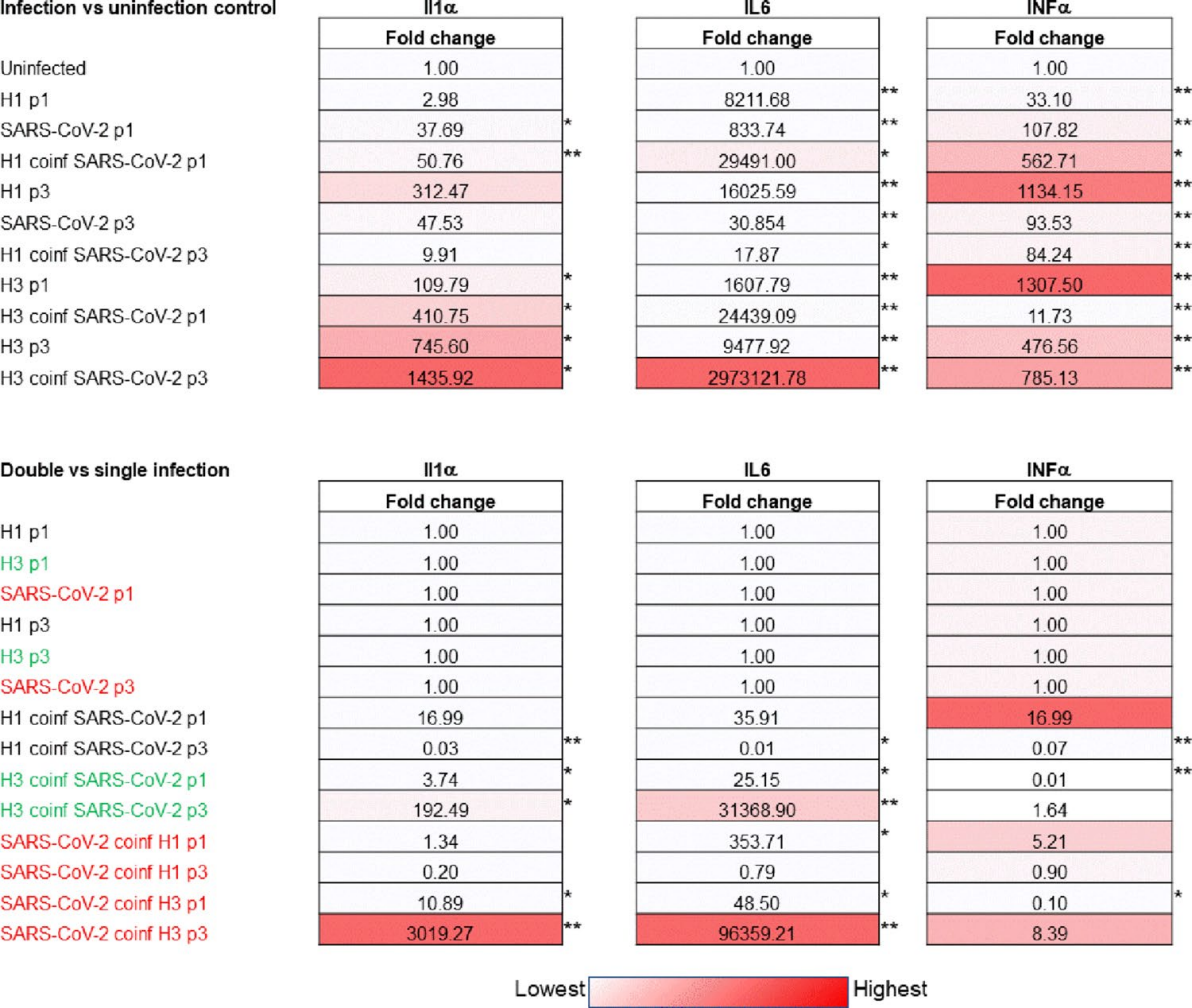
Expression of cellular innate immune response gene transcription during different passages on A549 cells. A549 cells were infected with the throat swab as indicated in Fig. 1. Infected cells were collected after 3 passages at 72 h post infection and used to extract RNA. One hundred nanograms of total RNA were amplified using primer and probes specific for interleukin-1 (IL-1α), interferon-alpha (IFN-α) and interleukin-6 (IL-6). The expression levels of interleukin-1 (IL-1α), interferon-alpha (IFN-α) and interleukin-6 (IL-6) mRNAs as indicators of cellular innate immune response were measured at 72 h post infection. Expression of the selected genes in three independent infected cultures was displayed as fold change relative to mock-infected cultures (uninfected) and cell infected with single virus using ΔΔCt method. The expression of target genes was normalized to the expression of 18 S gene. Values shown are mean obtained in 3 independent experiments. Cytokine mRNA expression between coinfection and single infection with significant difference is reported (*p < 0.05, **p < 0.01, *student’s t-test*).

The replicative experiments described above seem to assert that even in the absence of productive replication SARS-CoV-2 could acts as a counteracting factor for the passage of influenza virus concomitant present in the respiratory sample used for virus isolation. Thus, it was considered of interest examined the immunological response to the infection. To do this, it was chosen to examine the cytokine mRNA profile (IL-1α, IFN-α and IL-6 mRNA expression) at the first passage (p1) and at third passage (p3) in A549 cells (Fig. 2). Evaluating mRNA expression of the three cytokines it was evident a quite constant up regulation during infections with throat swab viral single and double positive compared to uninfected culture at passage 1 and passage 3 (Fig. 2).

Examining the mRNA cytokine expression comparing infection at passages 1 with a double versus single viral positive sample (samples 1 and 2 vs. samples 3, 4 and 5), infection with samples contains H3N2/SARS-CoV-2 compared to that with H3N2 alone and samples contains SARS-CoV-2/H3N2 compared to samples with SARS-CoV-2 alone exhibited a reduction of INF-α mRNA expression. The other combination with a double sample at passage 1 showed an up regulation for the three cytokines (Fig. 2). When examined the cytokine expression at passage 3, it was observed a significantly reduction of the three (IL-1α, INF−α, IL-6) cytokines expression in throat swab contains H1N1pdm09/SARS-CoV-2 compared to samples with H1N1pdm09 alone. Again, the other combination with a double sample at passage 3 showed an up regulation for the three cytokines (Fig. 2).

## Conclusion

In this study it was observed that during infection with a respiratory sample with a single viral positivity viral replication was higher than observed using a sample double positive to each specific single virus in all cell types used. In particular, using a throat swab double positive for H3N2/SARS-CoV-2 the replication activity of SARS-CoV-2 in MDCK and A549 cells and that of H3N2 in VERO E6 cells was totally impaired. In this context, the hampered SARS-CoV-2 replication, observed using the single positive throat swab infection, confirmed the SARS-CoV-2 ineffectiveness to replication in MDCK cells. These data suggest that even in the absence of a replicative activity the presence of SARS-CoV-2 in double positive samples attenuates influenza viral replication in MDCK cells. Of note, data on A549 cells were not entirely in agreement with the data obtained in in vitro experiments performed in our laboratory using different dose of coinfected SARS-CoV-2 and H1N1pdm09 virus showing an influenza virus increased replication in presence of SARS-CoV-2 coinfection [9]. Numerous studies in vivo have not always reported increased replication or worsened infection outcome during co-infection with SARS-CoV-2 and influenza viruses [10, 11]. However, the difference in viral replication observed in current study using double positive samples, compared to that with single positivity, could depend on the different infectious viral dose present in the throat swab used for infection [12]. Thus, although ct values were normalized to be similar, the throat samples double positive samples could contain reduced infectious viral particles compared to the single positive samples. An interesting hypothesis to be proved for the relevance of this phenomenon in the outcome of infection, it could be that during coinfection the interaction of each virus induce a high number of defective viral particle compared to single infection.

Examining the cytokine expression, an increased level of mRNA expression of the cytokine IL-1α, IL-6 and IFN-α was observed during each single and dual infection, at passage 1 and 3, compared to mock-infected culture. Furthermore, in general throat swab with H1N1pdm09/SARS-CoV-2 double positivity compared to H1N1pdm09 and SARS-CoV-2 single positivity showed a reduction in expression for all cytokines analyzed at passage 3. This data confirms the same profile of a reduced level of mRNA expression of the cytokines IL-1α, IL-6 and IFN-α observed in previous our study compared co-infection H1N1pdm09/SARS-CoV-2 to the single H1N1pdm09 or SARS-CoV-2 infection [9]. On the contrary, infection with H3N2/SARS-CoV-2 compared to H3N2 single infection showed an increase in expression for IL-1α and IL-6 but not for IFN-α in both passages as well as compared to SARS-CoV-2 single infection. Thus, it seems that H1N1pdm09 and H3N2 influenza virus coinfection with SARS-CoV-2 may exert a different type dependent effect on the cytokine profile with a potential role on the virus isolation outcome.

To date, several studies have reported that co-infection with influenza virus, SARS-CoV-2 and respiratory syncytial virus is recurrent [4, 13–15]. In the context of influenza virus / SARS-CoV-2 co-infection it has been reported opposite findings regarding the severity of disease [4, 16–18]. The data reported here have a limitation derived from the use of only two samples with a coinfection positivity. However, the limitation derived from the presence of scanty coinfection during the seasonal circulation of respiratory viruses. Improving this investigation should be regarded with interest to make clear the role of a double coinfection in the viral laboratory isolation techniques. Additionally, investigate other cytokine can more accurately shed light the role of inflammatory response. Thus, for the relevance of isolate and propagate influenza virus and SARS-CoV-2 to monitor virus strain in circulation, the interference in their isolation procedures have to be taken in to account.

## Data Availability

The data that support the findings of this study are all included in this article.

## Abbreviations

SARS-CoV-2: Severe acute respiratory syndrome coronavirus 2
MDCK: Madin-Darby canine kidney
VERO E6: African green monkey kidney epithelial cells
A549: Adenocarcinomic human alveolar basal epithelial cells
MEM: Minimum Essential Medium
DMEM: Dulbecco’s Modified Eagle Medium
FBS: Fetal bovine serum
BSL3: Biosafety level-3
IFN-α: Interferon-alpha
IL-1α: Interleukin 1 alpha
IL-6: Interleukin-6
